# Differences in Baseline Characteristics and Access to Treatment of Newly Diagnosed Patients With IPF in the EMPIRE Countries

**DOI:** 10.3389/fmed.2021.729203

**Published:** 2021-12-23

**Authors:** Abigél Margit Kolonics-Farkas, Martina Šterclová, Nesrin Mogulkoc, Katarzyna Lewandowska, Veronika Müller, Marta Hájková, Mordechai Kramer, Dragana Jovanovic, Jasna Tekavec-Trkanjec, Michael Studnicka, Natalia Stoeva, Simona Littnerová, Martina Vašáková

**Affiliations:** ^1^Department of Pulmonology, Semmelweis University, Budapest, Hungary; ^2^Department of Respiratory Diseases of the First Faculty of Medicine Charles University, University Thomayer Hospital, Prague, Czechia; ^3^Department of Pulmonary Medicine, Ege University Medical School, Izmir, Turkey; ^4^First Department of Pulmonary Diseases, Institute of Tuberculosis and Lung Diseases, Warsaw, Poland; ^5^Clinic of Pneumology and Phthisiology, University Hospital Bratislava, Bratislava, Slovakia; ^6^Rabin Medical Center, Institute of Pulmonary Medicine, Petah Tikva, Israel; ^7^Internal Medicine Clinic “Akta Medica”, Belgrade, Serbia; ^8^Pulmonary Department, University Hospital Dubrava, Zagreb, Croatia; ^9^Clinical Research Centre Salzburg, Salzburg, Austria; ^10^Tokuda Hospital Sofia, Sofia, Bulgaria; ^11^Faculty of Medicine, Institute of Biostatistics and Analyses, Masaryk University, Brno, Czechia

**Keywords:** IPF, treatment, regional accessibility, registry analysis, Central—Eastern Europe

## Abstract

Idiopathic pulmonary fibrosis (IPF) is a rare lung disease with poor prognosis. The diagnosis and treatment possibilities are dependent on the health systems of countries. Hence, comparison among countries is difficult due to data heterogeneity. Our aim was to analyse patients with IPF in Central and Eastern Europe using the uniform data from the European Multipartner IPF registry (EMPIRE), which at the time of analysis involved 10 countries. Newly diagnosed IPF patients (*N* = 2,492, between March 6, 2012 and May 12, 2020) from Czech Republic (*N* = 971, 39.0%), Turkey (*N* = 505, 20.3%), Poland (*N* = 285, 11.4%), Hungary (*N* = 216, 8.7%), Slovakia (*N* = 149, 6.0%), Israel (*N* = 120, 4.8%), Serbia (*N* = 95, 3.8%), Croatia (*N* = 87, 3.5%), Austria (*N* = 55, 2.2%), and Bulgaria (*N* = 9, 0.4%) were included, and Macedonia, while a member of the registry, was excluded from this analysis due to low number of cases (*N* = 5) at this timepoint. Baseline characteristics, smoking habit, comorbidities, lung function values, CO diffusion capacity, high-resolution CT (HRCT) pattern, and treatment data were analysed. Patients were significantly older in Austria than in the Czech Republic, Turkey, Hungary, Slovakia, Israel, and Serbia. Ever smokers were most common in Croatia (84.1%) and least frequent in Serbia (39.2%) and Slovakia (42.6%). The baseline forced vital capacity (FVC) was >80% in 44.6% of the patients, between 50 and 80% in 49.3%, and <50% in 6.1%. Most IPF patients with FVC >80% were registered in Poland (63%), while the least in Israel (25%). A typical usual interstitial pneumonia (UIP) pattern was present in 67.6% of all patients, ranging from 43.5% (Austria) to 77.2% (Poland). The majority of patients received antifibrotic therapy (64.5%); 37.4% used pirfenidone (range 7.4–39.8% between countries); and 34.9% nintedanib (range 12.6–56.0% between countries) treatment. In 6.8% of the cases, a therapy switch was initiated between the 2 antifibrotic agents. Significant differences in IPF patient characteristics and access to antifibrotic therapies exist in EMPIRE countries, which needs further investigation and strategies to improve and harmonize patient care and therapy availability in this region.

## Introduction

Idiopathic pulmonary fibrosis (IPF) is a rare, chronic, progressive, fibrotic lung disease associated with poor prognosis and high mortality (1–3). The median survival is between 2 and 5 years (1). Despite the largely undefined etiology, several exogenous environmental, and microbial factors seem to play key roles in the disease (4–7). The natural course of the disease is variable, and the factors that influence disease progression are unknown at an individual level (8).

The incidence of IPF has risen over time, it is between 3 and 9 cases per 1,00,000 per year (9). Regarding the systematic review of J. Hutchinson et al., there is a high variety in incidence and mortality rates depending on the geographic region (9). The overall prevalence of IPF is estimated at 30.2 cases per 1,00,000 (10).

Diagnosis and treatment possibilities of IPF are dependent on the health systems of countries as confirmed by several previous studies (11–13). Healthcare systems deal differently with diagnostic possibilities and availability. Considering treatment, expensive therapies are often introduced later as in wealthier countries and might be limited to a selected population of IPF (14, 15). Many off-label treatments are applied in rare diseases with potentially serious side effects (16).

Uniformity in diagnosis and treatment is crucial to patients dealing with persistent symptoms and uncertainty about the prognosis of their disease with a great impact on their quality of life (17). IPF has a considerable impact on the lives of the patients and the healthcare system (18). Medical professionals play an important role in the care of patients with IPF through patient education, monitoring medication compliance and safety, ensuring optimized medications for comorbidities, and preventive strategies. Patient education and counseling play key role in the shared decision-making model and are necessary for the management of this chronic disease (19).

Patient registries are organized systems that use observational study methods to collect uniform data (clinical and other) to evaluate specified outcomes for a population defined by a particular disease, condition, or exposure, and serve predetermined scientific, clinical, or policy purpose(s). Studies derived from well-designed and well-performed patient registries can provide a real-world view of clinical practice, patient outcomes, safety, and clinical comparative and cost-effectiveness analyses, and can serve as important tools for decision-making purposes (20–22). Comparison among countries is difficult due to data collection heterogeneity.

The aim of our study is to assess the baseline characteristics and treatment possibilities of patients with IPF in the same geographical—Central and Eastern Europe—region, by analysing the data of the European Multipartner IPF Registry (EMPIRE) countries (23).

## Patient Selection And Methods

### Study Design and Participants

The EMPIRE is a non-interventional, international, multicenter database of patients with IPF in Central and Eastern Europe (23). The objective of the registry is to evaluate the incidence, prevalence, and mortality of IPF in this area in Europe, and to determine the basic characteristics of patients with IPF. Another valuable outcome is the possibility of the comparison of different diagnostic and therapeutic differences among countries and assessment of the baseline characteristics of patients with IPF that participate in the EMPIRE using a uniform database platform.

Patients with IPF included in EMPIRE were diagnosed according to the American Thoracic Society/European Respiratory Society (ATS/ERS) consensus classification (1).

**Figure 1:**
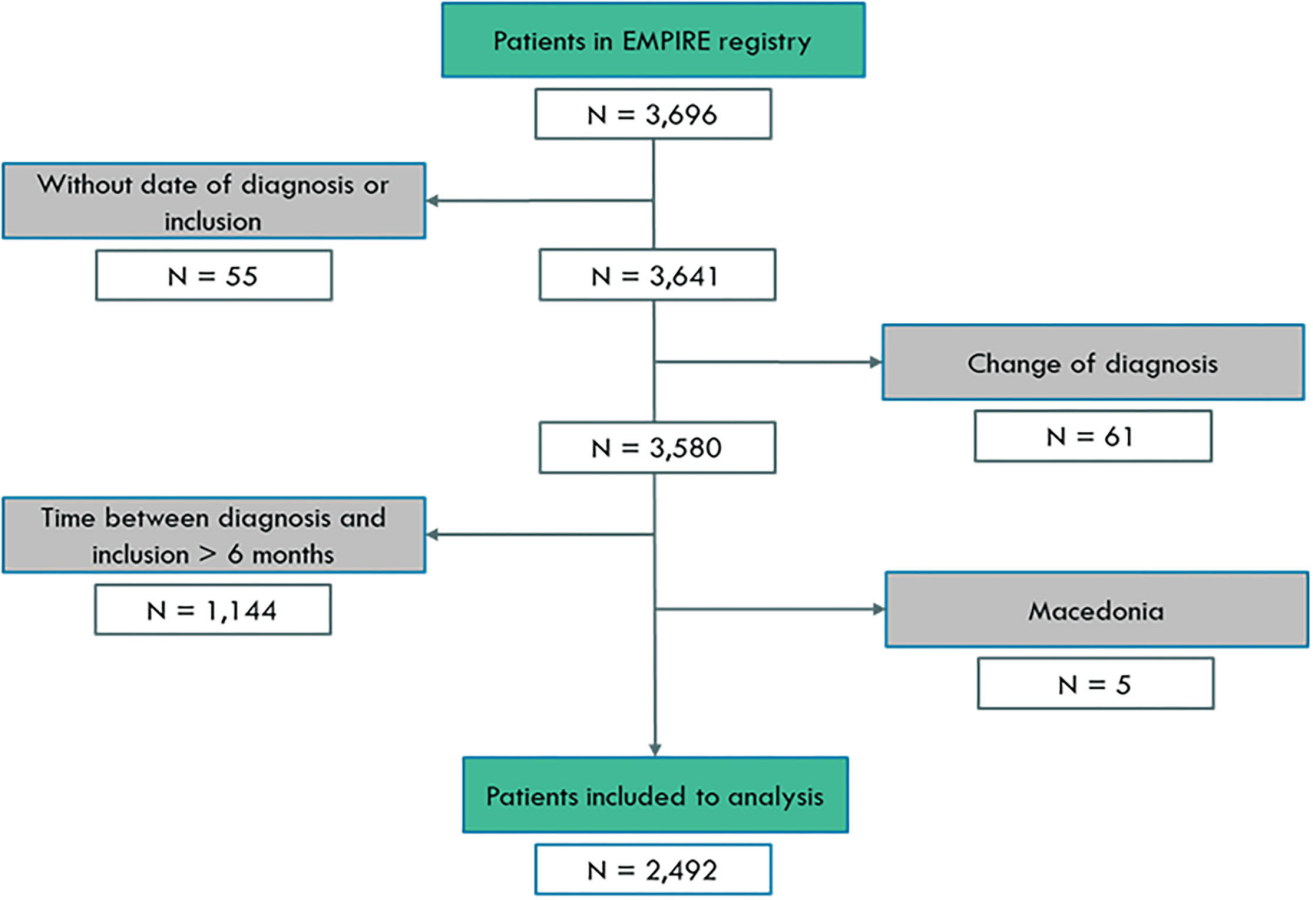
Patient selection for analysis.

All participants were included in the analysis from the EMPIRE registry between March 6, 2012 and May 12, 2020. Overall, 2,492 newly diagnosed patients were involved from 10 countries: Czech Republic (*N* = 971, 39.0%), Turkey (*N* = 505, 20.3%), Poland (*N* = 285, 11.4%), Hungary (*N* = 216, 8.7%), Slovakia (*N* = 149, 6.0%), Israel (*N* = 120, 4.8%), Serbia (*N* = 95, 3.8%,) Croatia (*N* = 87, 3.5%), Austria (*N* = 55, 2.2%), and Bulgaria (*N* = 9, 0.4%). The detailed patient selection process is shown in Figure 1.

Baseline characteristics, high-resolution CT (HRCT) pattern, and treatment data were analysed. Patient baseline demographics, including Gender-Age-Physiology (GAP) score, smoking history, symptoms, detailed lung function values [forced vital capacity (FVC), forced expiratory volume in 1 s (FEV1), total lung capacity (TLC), diffusing capacity of the lung for carbon monoxide (DLCO)], diffusing capacity for carbon monoxide (KLCO) and HRCT pattern were analyzed. In addition, information regarding comorbidities was obtained using chart reviews and was included in the analyses. Body mass index (BMI) and the 6-min walk test (6MWT) results were examined. Additionally, the number of patients in the respective groups was provided according to country (Table 1).

**Table 1:** Patient characteristics in individual countries.

	**Total** ***N* = 2,492**	**Czech Republic** ***N* = 971**	**Turkey** ***N* = 505**	**Poland** ***N* = 285**	**Hungary** ***N* = 216**	**Slovakia** ***N* = 149**	**Israel** ***N* = 120**	**Serbia** ***N* = 95**	**Croatia** ***N* = 87**	**Austria** ***N* = 55**	**Bulgaria** ***N* = 9**
**Median age, years (range)**											
All	2492/69 (54;82)	971/70 (54;82) T, S, R, A	505/68 (52;81) C, A	285/69 (57;84) S, R, A	216/70 (53;82) A	149/67 (48;79) C, P, A	120/67 (55;82) A	95/65 (48;79) C, P, A	87/70 (53;82) A	55/74 (63;87) C, T, P, HU, S, I, R, HR	9/69 (57;83)
Men	1786/69 (54;82)	719/70 (54;82)	383/68 (51;79)	206/69 (57;84)	125/69 (53;82)	97/68 (50;78)	83/69 (57;82)	57/67 (50;79)	64/71 (54;83)	45/74 (64;87)	7/71 (57;83)
Women	706/68 (54;82)	252/71 (54;82)	122/68 (54;83)	79/70 (57;84)	91/70 (54;82)	52/67 (40;81)	37/64 (50;78)	38/63 (44;81)	23/69 (51;76)	10/69 (62;81)	2/69 (68;69)
**Sex**, ***N*** **(%)**											
Men	1786 (71.7%)	719 (74.0%)	383 (75.8%)	206 (72.3%)	125 (57.9%)	97 (65.1%)	83 (69.2%)	57 (60.0%)	64 (73.6%)	45 (81.8%)	7 (77.8%)
Women	706 (28.3%)	252 (26.0%) HU	122 (24.2%) HU	79 (27.7%) HU	91 (42.1%) C, T, P	52 (34.9%)	37 (30.8%)	38 (40.0%)	23 (26.4%)	10 (18.2%)	2 (22.2%)
**Smoking**, ***N*** **(%)**											
Never-smoker	919 (37.1%)	395 (40.7%) T, P, R, HR	155 (30.7%) C, S, R	70 (24.6%) C, HU, S, R	90 (44.3%) P, HR	81 (55.1%) T, P, HR, A	50 (41.7%) HR	53 (56.4%) C, T, P, HR, A	12 (13.8%) C, HU, S, I, R	11 (20.0%) S, R	2 (22.2%)
Ever-smoker	1496 (60.4%)	562 (57.9%)	336 (66.5%)	206 (72.5%)	106 (52.2%)	62 (42.2%)	66 (55.0%)	36 (38.3%)	73 (83.9%)	42 (76.4%)	7 (77.8%)
Current smoker	60 (2.4%)	14 (1.4%)	14 (2.8%)	8 (2.8%)	7 (3.4%)	4 (2.7%)	4 (3.3%)	5 (5.3%)	2 (2.3%)	2 (3.6%)	0 (0.0%)
**BMI, kg/m**^**2**^ **(range)**	2443/28.0 (21.7;36.0)	967/28.6 (22.2;36.1) T, R, A	496/27.7 (21.3;34.9) C, R	281/28.0 (22.8;35.9) R	187/27.6 (20.8;37.7)	146/28.1 (22.2;37.1) R	120/27.7 (20.7;36.8)	95/26.1 (21.0;32.0) C, T, P, S	87/27.4 (21.5;34.0)	55/26.4 (21.5;34.2) C	9/29.2 (23.5;35.8)
**Dyspnea—NYHA**											
I	113 (4.9%)	13 (1.4%) T, P, HU, I, R, HR, A	23 (4.7%) C, P, HU, S, I, R, HR, A	20 (8.3%) C, T, S	31 (16.1%) C, T, S	0 (0.0%) T, P, HU, R, HR, A, M	9 (7.6%) C, T	4 (4.8%) C, T, S	8 (9.9%) C, T, S	5 (11.1%) C, T, S	0 (0.0%)
II	1325 (57.1%)	582 (62.7%)	172 (35.5%)	159 (66.3%)	106 (54.9%)	96 (68.6%)	73 (61.9%)	49 (59.0%)	56 (69.1%)	28 (62.2%)	4 (44.4%)
III	848 (36.5%)	325 (35.0%)	285 (58.8%)	55 (22.9%)	53 (27.5%)	44 (31.4%)	33 (28.0%)	22 (26.5%)	15 (18.5%)	11 (24.4%)	5 (55.6%)
IV	36 (1.6%)	8 (0.9%)	5 (1.0%)	6 (2.5%)	3 (1.6%)	0 (0.0%)	3 (2.5%)	8 (9.6%)	2 (2.5%)	1 (2.2%)	0 (0.0%)
**Cough**, ***N*** **(%)**											
Yes	1,594 (68.0%)	664 (73.0%) S	335 (66.7%) S	180 (64.7%)	118 (65.6%)	69 (51.1%) C, T, R	77 (68.8%)	60 (75.9%) S	53 (60.9%)	31 (57.4%)	7 (87.5%)
Dry	966 (60.6%)	459 (69.1%) T, I, A, B	175 (52.2%) C, A, B	106 (58.9%) A, B	64 (54.2%) A, B	50 (72.5%) A, B	33 (42.9%) C, A, B	42 (70.0%) A, B	25 (47.2%) A, B	8 (25.8%) C, T, P, HU, S, I, R, HR	4 (57.1%) C, T, P, HU, S, I, R, HR
Productive	599 (37.6%)	195 (29.4%)	159 (47.5%)	73 (40.6%)	53 (44.9%)	19 (27.5%)	43 (55.8%)	18 (30.0%)	28 (52.8%)	11 (35.5%)	0 (0.0%)
Unknown	29 (1.8%)	10 (1.5%)	1 (0.3%)	1 (0.6%)	1 (0.8%)	0 (0.0%)	1 (1.3%)	0 (0.0%)	0 (0.0%)	12 (38.7%)	3 (42.9%)
**Crackles**, ***N*** **(%)**	2254 (90.7%)	947 (97.5%) T, P, HU, S, R, A	392 (77.6%) C, P, HU, I, HR	264 (93.0%) C, T	192 (91.0%) C, T	127 (85.2%) C	112 (93.3%) T	83 (87.4%) C	84 (96.6%) T	44 (80.0%) C	9 (100.0%)
**Finger clubbing**, ***N*** **(%)**	874 (35.2%)	423 (43.6%) T, P, S, A	135 (26.7%) C, I, HR	70 (24.6%) C, HU, I, HR	81 (38.6%) P, S, A	26 (17.4%) C, HU, I, HR, B	55 (45.8%) T, P, S, A	27 (28.4%) HR	47 (54.0%) T, P, S, R, A	4 (7.3%) C, HU, I, HR, B, M	6 (66.7%) S, A
**GAP Score**, ***N*** **(%)**											
I	897(45.0%)	331 (42.1%)	163 (43.8%)	130 (53.5%)	83 (55.3%)	76 (58.5%)	38 (35.8%)	25 (38.5%)	31 (39.7%)	17 (31.5%)	3 (37.5%)
II	904 (45.4%)	380 (48.3%)	164 (44.1%)	97 (39.9%)	57 (38.0%)	46 (35.4%)	56 (52.8%)	33 (50.8%)	42 (53.8%)	27 (50.0%)	2 (25.0%)
III	192 (9.6%)	76 (9.7%)	45 (12.1%)	16 (6.6%)	10 (6.7%)	8 (6.2%)	12 (11.3%)	7 (10.8%)	5 (6.4%)	10 (18.5%)	3 (37.5%)
**HRCT pattern**, ***N*** **(%)**											
UIP	1523 (67.5%)	647 (73.8%) T, HU, S, R, A	284 (62.1%) C, P, A	207 (77.2%) T, HU, S, R, A	119 (58.3%) C, P	76 (56.3%) C, P	75 (76.5%) R, A	42 (49.4%) C, P, I	48 (61.5%)	19 (43.2%) C, T, P, I	6 (66.7%)
Possible UIP	653 (29.0%)	218 (24.9%)	138 (30.2%)	60 (22.4%)	78 (38.2%)	53 (39.3%)	19 (19.4%)	32 (37.6%)	27 (34.6%)	25 (56.8%)	3 (33.3%)
Inconsistent with UIP	79 (3.5%)	12 (1.4%)	35 (7.7%)	1 (0.4%)	7 (3.4%)	6 (4.4%)	4 (4.1%)	11 (12.9%)	3 (3.8%)	0 (0.0%)	0 (0.0%)
**Comorbidities**											
0	211 (8.5%)	77 (7.9%) P, HU, S, I, R, M	29 (5.7%) P, HU, S, I, R, M	27 (9.5%) C, T, I, R, HR, M	32 (14.8%) C, T, I, HR, M	24 (16.1%) C, T, I, HR, M	0 (0.0%) C, T, P, HU, S, R, A, B, M	16 (16.8%) C, T, P, I, HR, M	2 (2.3%) P, HU, S, R, A, M	3 (5.5%) I, HR, M	1 (11.1%) I
1	449 (18.0%)	144 (14.8%)	73 (14.5%)	65 (22.8%)	55 (25.5%)	45 (30.2%)	5 (4.2%)	35 (36.8%)	8 (9.2%)	15 (27.3%)	4 (44.4%)
2	463 (18.6%)	179 (18.4%)	94 (18.6%)	63 (22.1%)	43 (19.9%)	27 (18.1%)	10 (8.3%)	25 (26.3%)	8 (9.2%)	13 (23.6%)	1 (11.1%)
>2	1369 (54.9%)	571 (58.8%)	309 (61.2%)	130 (45.6%)	86 (39.8%)	53 (35.6%)	105 (87.5%)	19 (20.0%)	69 (79.3%)	24 (43.6%)	3 (33.3%)

*Data are N (%) or median (range); GAP, Gender-Age-Physiology*.

The study was performed in accordance with the Declaration of Helsinki and ethical approval was obtained in each country according to respective regulations.

### Statistical Analysis

The study aimed to evaluate the differences and/or similarities in clinical data and treatment in patients with IPF in Central and Eastern Europe. A descriptive statistical analysis was performed and included absolute and relative frequencies for categorical variables and medians, with 5th−95th percentile ranges calculated for quantitative variables (in plots that were completed with interquartile range [IQR]). Significant differences among groups were analysed using the χ^2^-test for categorical variables and Kruskal–Wallis tests for quantitative variables. If differences were statistically significant, *post-hoc* testing with a Bonferroni correction was used to identify homogeneous groups. The level of statistical significance was set at *p* < 0.05. Analyses were performed using SPSS v25 (IBM Corporation, Armonk, NY, USA) and Stata 14.2. (StataCorp., Lakeway Drive, TX, USA).

## Results

### Patient Characteristics

Overall, 3,696 patients with IPF participated in the study. Information about the enrollment is shown in Figure 1. The final analysis included 2,492 patients. Exclusion of patients where the time of diagnosis and inclusion was over 6 months represented prevalent cases and not incident cases. To analyse the longitudinal outcome, newly diagnosed patients were included in the registry, defined by <6 months between inclusion and diagnosis. Participants with no date of inclusion in the study (*N* = 55) or with an inclusion time that had been more than 6 months compared with the time of diagnosis (*N* = 1,144) or who had a change in diagnosis (*N* = 61) were excluded from the analysis.

Information on EMPIRE member distribution is summarized in Table 1. Patients with the highest average age came from Austria; Austrian IPF patients were typically older than patients from most of the other countries. Patients from Serbia were the youngest and appeared to be significantly younger than participants from the Czech Republic, Poland, and Austria. Patients with IPF were more frequently men, and a significantly higher ratio of women was noted in Hungary as in the Czech Republic, Turkey, and Poland. The highest percentile contribution of men was noted in Bulgaria and Austria. In almost every country, more than 50% of patients had a smoking history. Across all countries, patients in Croatia had the highest ratio of patients with a history of smoking, whereas this number was the lowest in Serbia. BMI had the highest average value in Bulgaria, followed by the Czech Republic, and the lowest in Serbia. New York Heart Association (NYHA) class IV dyspnea was very rare among the patients; most frequently, NYHA class II dyspnea occurred, and it was most common among the Slovakian patients. Cough was present in more than two-thirds of the cases; patients in Serbia and Bulgaria complained about it in most of the cases. Dry cough was more typical than productive cough in every country. Crackles were present in more than 90% of the cases with the highest ratio in the Czech Republic and Bulgaria.

GAP scores I and II had almost the same frequency among all countries and together they accounted for more than 90% of the cases. Slovakian patients had GAP score I most frequently, GAP score II was mostly observable in Croatia, while GAP score III was most common in Bulgaria and Austria.

HRCT lung imaging was described according to the ATS/ERS consensus classification in all patients (1). Usual interstitial pneumonia (UIP) pattern was present in approximately two-thirds of the patients with the highest prevalence in Poland. A possible UIP pattern was the most frequent in Austria, whereas a pattern inconsistent with UIP was most common in Serbia.

### Analysis of Lung Function

Baseline lung function values are summarized in Table 2. FVC was between 50 and 80% in 49.3% and >80% in 49.3% of the patients. Most IPF-patients with FVC > 80% were registered in Poland, while the lowest number frequency was in Israel. Baseline FEV1% predicted was between 70% and 90% in 40.1% of the cases and >90% in 32.8% of the patients. Most cases with FEV1% > 90% were registered in Slovakia and Poland, while the lowest was in Israel. FEV1/FVC was between 70% and 80% in 22.3%, >80% in 70.6%, and <70% in 7.1% of the patients at the time of enrollment. Most patients with FEV1/FVC > 80% were registered in Slovakia and the highest number of patients with FEV1/FVC <70% values came from Austria (20%). TLC% predicted had the highest average value in Poland and Slovakia, while the lowest average value in Israel. DLCO% and KLCO% predicted values were the highest in Hungary and the lowest in Serbia. Patients from Slovakia had the biggest average distance of 6MWT, whereas this value was the lowest in the Czech Republic.

**Table 2:** Lung function values and 6-min walk test in individual countries.

**Valid *N*/median** **(5th;95th percentile)**	**Total** ***N* = 2,497**	**Czech Republic** ***N* = 971**	**Turkey** ***N* = 505**	**Poland** ***N* = 285**	**Hungary** ***N* = 216**	**Slovakia** ***N* = 149**	**Israel** ***N* = 120**	**Serbia** ***N* = 95**	**Croatia** ***N* = 87**	**Austria** ***N* = 55**	**Bulgaria** ***N* = 9**
FVC (L)	2293/2.59 (1.36;4.10)	911/2.56 (1.45;3.91) T, P, I	454/2.37 (1.19;3.87) C, P, S, I, HR, A	271/2.92 (1.61;4.54) C, T, HU, I	189/2.35 (1.29;4.05) P, S, HR	131/2.83 (1.55;4.35) T, HU, I	114/1.96 (0.91;3.53) C, T, P, S, R, HR, A	73/2.70 (1.37;4.15) I	87/2.79 (1.53;4.33) T, HU, I	55/2.68 (1.68;4.43) T, I,	8/2.57 (1.43;3.66)
FVC (% predicted)	2267/77 (48;114)	910/76 (50;106) P, S, I, HR	450/74 (45;110) P, S, I, HR	271/87 (59;127) C, T, HU, I	168/76 (43;115) P, S, I	131/85 (52;121) C, T, HU, I	114/63 (34;104) C, T, P, HU, S, R, HR, A	73/81 (47;115) I	87/86 (52;123) C, T, I	55/84 (49;120) I	8/76 (42;115)
FEV1 (L)	2286/2.14 (1.16;3.31)	910/2.20 (1.27;3.27) T, HU, I	451/1.96 (1.03;3.08) C, P, S, I, R	270/2.32 (1.33;3.62) T, HU, I	186/1.97 (1.15;3.32) C, P, S, I	132/2.41 (1.38;3.68) T, HU, I	114/1.71 (0.82;2.90) C, T, P, HU, S, R, HR, A	73/2.34 (1.22;3.54) T, I	87/2.18 (1.28;3.23) I	55/2.26 (1.28;3.35) I	8/2.19 (1.04;2.92)
FEV1 (% predicted)	2258/81 (51;114)	909/81 (55;110) T, P, S, I	448/77 (48;110) C, P, S, I	268/89 (59;122) C, T, HU, I	165/79 (45;115) P, S, I	132/89 (57;124) C, T, HU, I	114/70 (39;103) C, T, P, HU, S, R, HR, A	73/84 (50;115) I	87/81 (55;113) I	54/85 (43;107) I	8/78 (46;110)
FEV1/FVC	2274/84 (68; 97)	900/86 (71; 98) T, P, HR, A	457/83 (70; 96) C, P, HR, AT	270/81 (65; 91) C, T, HU, S, I, R	184/84 (70; 95) P, HR, A	130/85 (68; 96) P, HR, A	114/86 (68; 97) P, HR, A	70/85 (69; 99) P, HR, A	87/78 (56; 94) C, T, HU, S, I, R	54/79 (52; 91) C, T, HU, S, I, R	8/81 (73; 91)
TLC (L)	1984/4.23 (2.21;6.60)	853/4.28 (2.62;6.51) T, S, I, A	280/3.85 (2.08;5.89) C, P, S, A	233/4.67 (0.00;6.98) T, HU, I	181/3.96 (2.11;6.32) P, S, A	124/4.68 (3.06;7.76) C, T, HU, I, R	105/3.77 (2.13;6.16) C, P, S, A	71/4.23 (0.00;6.50) S, A	82/4.19 (2.42;6.92)	55/4.77 (3.20;6.72) C, T, HU, I, R	0/0
TLC (% predicted)	1963/70 (41;100)	854/69 (46;97) T, P, S	279/64 (43;95) C, P, S, A	231/78 (0;109) C, T, HU, I	162/67 (38;100) P, S	124/78 (54;151) C, T, HU, I, R, HR	105/62 (44;92) P, S, A	71/67 (0;100) S	82/69 (45;98) S	55/76 (52;108) T, I	0/0
DLCO%	2126/46.8 (0.0;80.5)	895/46.4 (23.7;73.0) HU, R	384/46.1 (0.0;80.7) HU, R	250/47.9 (0.0;86.6) HU, R	149/59 (24;104) C, T, P, S, I, R, HR, A	130/51 (0;78) HU, R	107/45.4 (20.6;87.0) HU, R	70/30.2 (0.0;59.2) C, T, P, HU, S, I, HR, A	79/42.2 (9.2;72.3) HU, R	54/45.9 (0.0;72.9) HU, R	8/35.6 (19.4;69.7)
KLCO%	2041/75 (0;119)	850/76 (13;115) P, HU, I, R, HR	388/77 (0;123) P, R	220/65 (0;105) C, T, HU, S	153/86 (14;140) C, P, I, R, HR, A	131/76 (0;176) P, R	88/67 (0;104) C, HU	73/53 (0;188) C, T, HU, S	81/65 (15;103) C, HU	54/73 (0;111) HU	0/0
6MWT Distance (m)	1231/390 (168;560)	274/360 (160;530) P, S	373/375 (135;511) P, S	189/420 (235;600) C, T, S	129/400 (170;578) S	72/495 (355;590) C, T, P, HU, I, R, HR	72/403 (90;540) S	39/400 (140;545) S	66/401 (190;540) S	17/460 (196;635)	0/0

In our study, the FVC% predicted values were tested in 91.8% of the total population. The highest ratio appeared in Croatia and Austria as patients in both countries underwent testing for FVC in 100% and the lowest ratio could be seen in Serbia (76.8%). FEV1% predicted was measured in all cases in Croatia (100%), whereas the lowest ratio of patients was in Hungary (76.4%). FEV1/FVC was calculated in most cases in Croatia and the least in Serbia. TLC% predicted evaluation had the highest percentage in Austria (100%), whereas, in Bulgaria, there was no evaluation of TLC% predicted. DLCO% predicted was entered into the registry with the highest patient participation in Austria (98.2%) and the lowest in Hungary (69.0%). KLCO% predicted testing ratios were the following: highest test proportion in Austria and no tested patient for KLCO% predicted in Bulgaria. 6MWT was performed in most cases in Croatia, while no 6MWT was done in the case of Bulgarian patients.

### Patient Comorbidities

**Figure 2:**
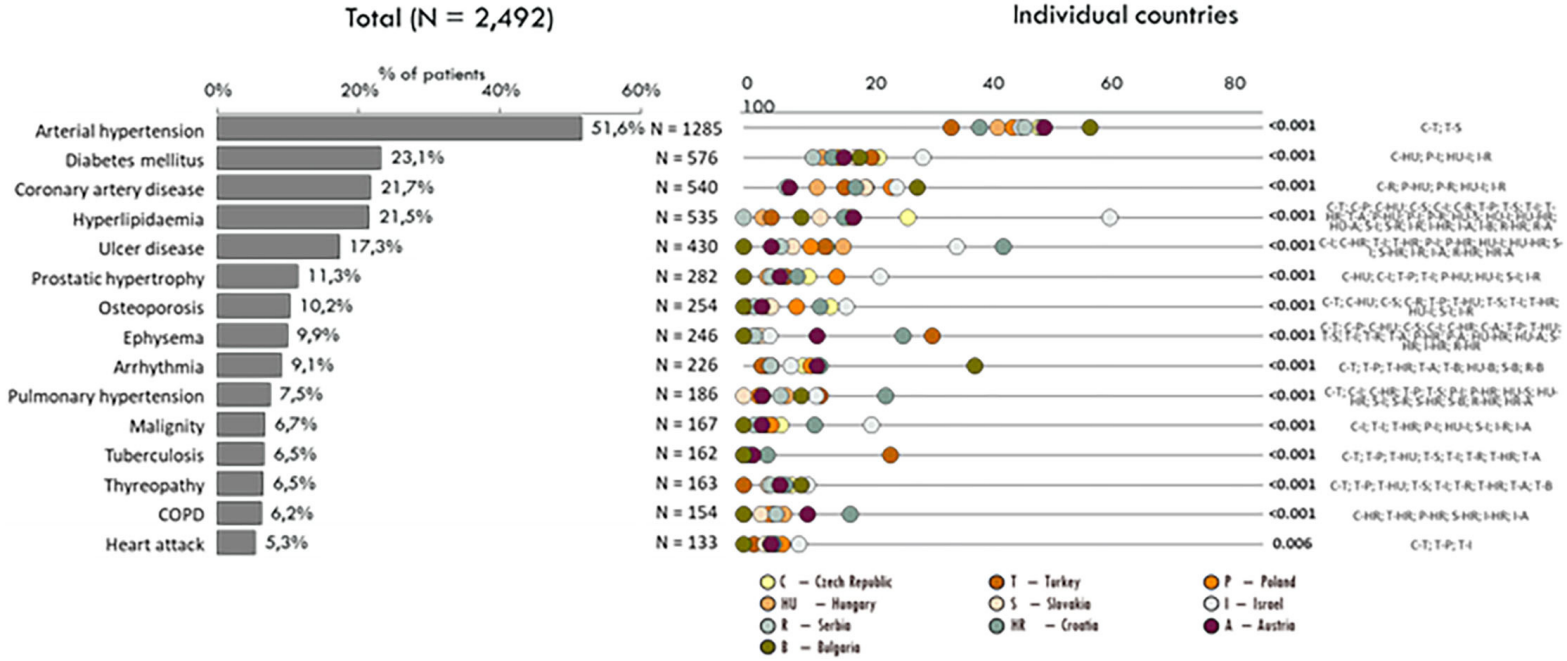
Comorbidities according to the countries.

Significant alterations were noted in comorbidities in the different countries. The leading comorbidities were cardiovascular diseases followed by gastrointestinal and pulmonary disorders. Overall, more than half of the patients had more than 2 comorbidities. In general, patients in Serbia had the lowest rate of comorbidities, whereas patients from Israel had a medical history with at least 2 co-occurring disorders. A detailed analysis of comorbidities is shown in Figure 2.

### Antifibrotic Treatment

More than 50% of the patients received antifibrotic therapy. Pirfenidone and nintedanib use showed significant differences between countries. The use of pirfenidone was the most frequent in Turkey; a significantly higher proportion of Turkish patients received pirfenidone at the time of investigation as compared with the other countries participating in the study. The application of nintedanib was most frequent in Hungary: more than half of the patients received it as antifibrotic treatment. The summary of antifibrotic treatment can be found in Table 3.

**Table 3:** Antifibrotic treatment in individual countries.

	**Total** ***N* = 2492**	**Czech Republic** ***N* = 971**	**Turkey** ***N* = 505**	**Poland** ***N* = 285**	**Hungary** ***N* = 216**	**Slovakia** ***N* = 149**	**Israel** ***N* = 120**	**Serbia** ***N* = 95**	**Croatia** ***N* = 87**	**Austria** ***N* = 55**	**Bulgaria** ***N* = 9**
Pirfenidone	750 (30.1%)	364 (37.5%) T, P, HU, S, I, R, HR, A	201 (39.8%) C, P, HU, S, I, A	73 (25.6%) C, T, HU, S, I, A	22 (10.2%) C, T, P, S, R, HR	11 (7.4%) C, T, P, HU, I, R, HR	20 (16.7%) C, T, P, S, R, HR	27 (28.4%) C, HU, S, I, A	25 (28.7%) C, HU, S, I, A	6 (10.9%) C, T, P, R, HR	1 (11.1%)
Nintedanib	689 (27.6%)	246 (25.3%)	72 (14.3%)	58 (20.4%)	121 (56.0%)	74 (49.7%)	52 (43.3%)	19 (20.0%)	11 (12.6%)	34 (61.8%)	2 (22.2%)
Switch	169 (6.8%)	94 (9.7%)	22 (4.4%)	8 (2.8%)	15 (6.9%)	0 (0.0%)	18 (15.0%)	3 (3.2%)	6 (6.9%)	3 (5.5%)	0 (0.0%)
None	884 (35.5%)	267 (27.5%)	210 (41.6%)	146 (51.2%)	58 (26.9%)	64 (43.0%)	30 (25.0%)	46 (48.4%)	45 (51.7%)	12 (21.8%)	6 (66.7%)

*Data are N (%) or median (range). Data are only expressed as absolute number of patients and corresponding proportion percentage*.

As the availability of different antifibrotics might be dependent on the healthcare provider regulation of the individual country, reimbursement, and country-specific regulations are described in Table 4.

**Table 4:** Antifibrotic treatment availability in individual countries.

**Country**	**Year of joining EMPIRE**	**Number of patients receiving antifibrotic treatment, *N* (% all patients in the given country)**	**Reimbursement specifics**
*Czech Republic*	2015 (2012–2015 as National Czech Registry of IPF)	• nintedanib: 246 (25.3) ∘ pirfenidone: 364 (37.5)	• 2015–2018 covered on individual request Reimbursed since 2018 in patients fulfilling predefined criteria covered by health insurance ∘ 2014–2017 covered on individual request Reimbursed since 2017 in patients fulfilling predefined criteria covered by health insurance
*Turkey*	2016	• nintedanib: 72 (14.3) ∘ pirfenidone: 201 (39.8)	• September 23, 2017 Nintedanib received a refund. Free for those with FVC more than 50%, DLCO more than 30%, <10% FVC loss in 6 months ∘ October 11, 2016–267 mg capsules and 200 mg tablets received a refund 01 April 2020–600 mg tablets received a refund September 9, 2020–267 mg tablets and 801 mg tablets received a refund. Free for those with FVC more than 50%, DLCO more than 30%, <10% FVC loss in 6 months
*Poland*	2015	• nintedanib: 58 (20.4) ∘ pirfenidone: 73 (25.6)	•2018 Therapeutic program (fully reimbursed in patients with: FVC ≥ 50% DLCO ≥ 30%). Stopping rule: decrease of 10% in FVC in first year of treatment and then in 6 months assessed every 6 months ∘ 2017 Therapeutic program (fully reimbursed in patients with: FVC ≥ 50% DLCO ≥ 30%) Stopping rule: decrease of 10% in FVC in first year of treatment and then in 6 months assessed every 6 months
*Hungary*	2015	• nintedanib: 121 (56.0) ∘ pirfenidone: 22 (10.2)	• 2015–2017: individual request coverage by national insurance Since 2017 according label fully covered by national insurance ∘ 2017: According label fully covered by national insurance
*Slovakia*	2015	• nintedanib: 74 (49.7) ∘ pirfenidone: 11 (7.4)	• Available since 2015 based on individual reimbursement ∘ Available since 2015 based on individual reimbursement
*Israel*	2018	• nintedanib: 52 (43.3) ∘ pirfenidone: 20 (16.7)	• 2014–2016: Compassionate use program 2016: Fully covered ∘ 2016: Fully covered
*Serbia*	2015	• nintedanib: 19 (20.0) ∘ pirfenidone: 27 (28.4)	• 2017: According label, not covered by national insurance, but at the cost of referral institutions (4 University hospitals of Pulmonology) based on decisions of their Consilia for Fibrosis ∘ 2016: For all cases of IPF, not covered by national insurance, but at the cost of referral institutions (4 University hospitals of Pulmonology) based on decisions of their Consilia for Fibrosis
*Croatia*	2016	• nintedanib: 11 (12.6) ∘ pirfenidone: 25 (28.7)	• 2017: Fully covered by National Health insurance fund for patients with FVC between 50% and 80% Stopping rule: decrease of FVC >10% at any time during 12 months Reassessment: every 12 months ∘ 2017: Fully covered by National Health insurance fund for patients with FVC between 50 and 80% Stopping rule: decrease of FVC >10% at any time during 12 months Reassessment: every 12 months
*Austria*	2018	• nintedanib: 34 (61.8) ∘ pirfenidone: 6 (10.9)	• Available since 2015, the access for patients is based on individual reimbursement. Full reimbursement for IPF no restrictions—systemic sclerosis/progressive fibrosing ILD individual reimbursement ∘ Available since 2011, only individual reimbursement for IPF with FVC ≥ 50 and ≤ 80 and stopping rule (10% in 6 months)—new indications still under discussion
*Bulgaria*	2018	• nintedanib: 2 (22.2) ∘ pirfenidone: 1 (11.1)	• Since April 2018 Reimbursed by National Health insurance fund for patients over 50 year old and with FVC between 50 and 80% and DLCO between 79 and 30%. Stopping rule for patients reached DLCO or FVC bellow lower limit Reassessment every 6 month ∘ Since April 2018 Reimbursed by National Health insurance fund for patients over 50 year old and with FVC between 50–80% and DLCO between 79 and 30%. Stopping rule for patients reached DLCO or FVC bellow lower limit Reassessment every 6 month

## Discussion

Our data are the first to compare intercountry differences in patients with IPF using the common platform of EMPIRE enabling uniform data input and analysis. While real-world registries have limitations, our results confirm profound differences in baseline characteristics, lung function, HRCT pattern, and comorbidities in the patients with IPF from 10 Central and Eastern European countries.

Maximizing the potential of precision medicine for patients and healthcare services is a major social challenge. Disease registries have great potential to provide insight into real-world data and, consequently, provide information for planning healthcare services (24, 25). With their help, it is easier to collect data about complaints, symptoms, and quality of life of the patients, to investigate the effects and adverse effects of different treatments and to evaluate the disease development. However, registry data may suffer from bias and vary between countries as a result of incomplete registration, precluding measurement of true incidence and prevalence (26). Previously, the European Respiratory Journal emphasized the importance of registry data in IPF: prospective cohorts mean a solution to support patient care and research in complex chronic diseases (26).

Data collected from clinical trials are often misleading due to selection bias. Globally, there are significant differences in the incidence, prevalence, diagnostic approach, therapies, and survival for patients with IPF according to continents and countries. For example, the prevalence of IPF varies widely depending on location, identifying criteria, and year of study, ranging from 3 to 6 per 1,00,000 in the United Kingdom up to 16–18 per 1,00,000 in Finland (27, 28). Individual registries, generally, differ from each other, thus there might be differences regarding inclusion criteria, frequency, and outcome of IPF exacerbations, comorbidities, genetic factors and variance, efficacy and safety of pharmaceutical therapy, predictors of outcome, etc. With international registries, it is possible to create large datasets that enable clinicians and researchers to compare regions, countries, and time periods. According to McCormick et al., who made a comparative analysis of Cystic Fibrosis Registry data from the United Kingdom with other countries, the development of national cystic fibrosis databases has enabled a comparison between countries in key clinical outcomes. However, the authors highlighted the limitation of the study and urged a standardization of data collection between national cystic fibrosis registries to obtain a greater understanding from international and intercontinental comparisons (29).

In this study, we present clinical data from EMPIRE, the registry of patients with IPF from Central and Eastern Europe (23). We evaluated patient baseline characteristics, clinical symptoms, radiological features, spirometric values, and therapeutic solutions to emphasize similarities and differences between 10 countries. Despite living in the same geographical area, there were statistically significant differences regarding all the examined features and parameters. However, through this study, similarities and main differences could be highlighted and the shortcomings in terms of uniformity can be improved in the future. Currently, there are 2 IPF-registries in which Central and Eastern Europe is a partaker, namely EMPIRE and eurIPFreg. There are 12 other IPF-registries in Europe, however, they only include patients from one country (24).

The quality of healthcare system of a country can be estimated, for example, by the proportion of the structured clinical examinations performed (30). While not comparable, clinical data from well-structured IPF national registries might give some hints about diversities in different countries. The national IPF-registry of Spain, the SEPAR National Registry analysed the data from 608 patients between 2012 and 2017 (31). The electronic registry of IPF in the United Kingdom, the UK IPF Registry has counted 2,474 registered patients in the time period of 2013–2019 (32). To the INSIGHTS-IPF registry of Germany, 588 patients were entered between 2012 and 2018 (33). Between 2012 and 2016, 647 patients were registered to the Australian Idiopathic Pulmonary Fibrosis Registry (AIPFR) (8). For example, dyspnea was less frequent in the UK IPF registry, in comparison with the other 4 registries. In AIPFR, better baseline lung function was noted than in the other cohorts. GAP stage I was the rarest in EMPIRE compared with the other 4 registries, while UIP HRCT pattern appeared more often in our analysis. Our data show comparable lung function values for the most published registry data.

The organization of detailed evidence is considered to be a very strict measure as its purpose is also to create clinical practice guidelines (34, 35). Clinical practice guidelines are by their nature general recommendations aimed for broad applicability in the clinical setting. The applicability, however, is limited by numerous factors. The challenge of using guidelines on daily basis is that these guidelines are likely to be disease-oriented and not patient-oriented. Guideline recommendations are mainly based on the disease severity without taking coexistent conditions and other factors (e.g., factors that are used by physicians to individualize diagnosis and treatment), into consideration (36). High-quality meta-analyses and systematic reviews of randomized control trials (RCTs) or RCTs with a very low risk of bias stand in first place on the hierarchy of levels of evidence from published papers (34, 37). RCTs are created to maximize internal validity by studying a strictly defined population in a controlled setting, hence, establishing the efficacy of treatment (36, 38). Their results may have limited applicability to patients in clinical settings (39). These trials generally register a thoroughly selected patient population that meets strict inclusion criteria and exclusion criteria, including regular laboratory and clinical monitoring and measure objective parameters of efficacy. In “real world” clinical practice, however, the patients are unselected, monitoring is likely to be less frequent, and effectiveness is the most relevant outcome (36, 40). Pragmatic trials and observational studies can play an important role in addition to RCTs as they are created to recreate conditions in the daily clinical practice (40). Observational studies examine large groups of patients to evaluate long-term outcomes, examine very important consequences, such as mortality, and examine outcomes that may not be easily assessed by RCTs (e.g., pharmacoeconomic data). Recent analyses of data gained by RCTs and observational studies concluded that the effects of treatment revealed in observational studies were not greater or qualitatively different from those of RCT comparing the same treatments (41, 42). The reliance on RTCs as the highest level of evidence is thus challenged (43). Although observational studies should not replace RCTs, they can be useful in complementing the results of such trials. Well-designed observational studies can identify clinically important differences among therapeutic options and provide information on long-term drug effectiveness and safety (39). As a result of a review that compared the two methods used in good clinical practice concluded that the development of country-specific guidelines or local guidelines for each region would provide more suitable practical solutions. Besides, factors, such as social factors and expenses—that influence choice of the patients—and therapy adherence would be better considered (36).

Randomized control trials play the leading role and are inevitable when developing and testing new pharmaceutical substances. Over the last years—despite being a rare disease—numerous large, multicenter RCTs have been conducted culminating in the approval of 2 drugs for the treatment of IPF (44).

Our data confirmed, that in IPF, significant differences exist in drug availability according to countries, possibly resulting from high costs when introducing new treatments. As we summarized data for nintedanib and pirfenidone, there were no two countries with the same policy for providing these drugs to patients. As a result, regional differences in survival might be observed due to treatment differences arising from national regulations. Comparisons of the effectivity of antifibrotics might be further challenged, as availability changes over time and over regions. For example, in Australia, antifibrotic treatment was available through clinical trials, special access programs, and private purchase by the time of inclusion in the published AIPFR document (8). Further studies are needed to evaluate the long-term outcome in patients treated with antifibrotics by stratifying cases according to already developed prognostic factors (45).

Healthcare specialists, patient organizations, and EU regulatory bodies should work to cease inequalities in patient care also highlighted in our data.

The limitation of our study is the disproportion in the number of patients from different countries, as it varied from 971 (Czech Republic) to 9 patients (Bulgaria) and 55 (Austria) mainly representing the time of being in the Registry. Differences in center size, the number of centers, time to enrollment and operator practice, and ethnic/cultural heterogeneity might all affect the outcome of the analysis.

## Conclusions

Well-organized and unified registries for patients with IPF are indispensable to achieve better outcomes. In this study, we proved significant differences in the characteristics of patients with IPF and described differences in availability to antifibrotic therapies in EMPIRE countries that needs further investigation and strategies to improve patient care in this region. Equal participation rates and complete data registration in EMPIRE are fundamental to maximize precision. Unified methods and maximal accuracy are key elements to better understanding and more effective treatment of IPF. Inequalities resulting from differences in the availability of antifibrotics should be managed with international cooperation.

## Data Availability Statement

The datasets presented in this article are not readily available because need to file individual inquery to EMPIRE headquarters. Requests to access the datasets should be directed to empire@iba.muni.cz.

## Ethics Statement

The studies involving human participants were reviewed and approved by the EMPIRE registry protocol was approved in each country by the respective Ethical Committee. Written informed consent was obtained from all individual patients, who were enrolled in accordance with the Helsinki Declaration. The patients/participants provided their written informed consent to participate in this study.

## Author Contributions

AK-F wrote the first draft of the manuscript. MŠte, NM, KL, VM, MH, MK, DJ, JT-T, MStu, NS, and MV contributed to conception and the design of the study. SL worked out the concept of statistical evaluation and performed statistical analysis. All authors contributed to the data collection, manuscript revision, read, and approved the submitted version.

## Funding

The EMPIRE registry and this investigator-initiated study have been supported with funding from Boehringer Ingelheim (BI) and F. Hoffman-La Roche (Roche). BI and Roche had no role in the study design, analysis, or interpretation of the results. BI and Roche were given the opportunity to review the manuscript for medical and scientific accuracy as it relates to BI and Roche substances and intellectual property considerations.

## Conflict of Interest

MH has received lecture fees and consulting fees and served as an advisory board member for Boehringer Ingelheim. VM has received consulting fees from Roche and Boehringer Ingelheim. DJ has received consulting fees or honorarium and payment for lectures from Roche and Boehringer Ingelheim. JT-T has received payment for lectures from Roche and Boehringer Ingelheim and consulting fees from Boehringer Ingelheim. MV has received an independent grant from Roche and consultancy, lecture, and advisory board fees from Boehringer Ingelheim and Roche. MS has received consultation fees from Boehringer Ingelheim. The remaining authors declare that the research was conducted in the absence of any commercial or financial relationships that could be construed as a potential conflict of interest.

## Publisher's Note

All claims expressed in this article are solely those of the authors and do not necessarily represent those of their affiliated organizations, or those of the publisher, the editors and the reviewers. Any product that may be evaluated in this article, or claim that may be made by its manufacturer, is not guaranteed or endorsed by the publisher.
